# Surfactant proteins, SP-A and SP-D, in respiratory fungal infections: their role in the inflammatory response

**DOI:** 10.1186/s12931-016-0385-9

**Published:** 2016-06-01

**Authors:** Laura Elena Carreto-Binaghi, El Moukhtar Aliouat, Maria Lucia Taylor

**Affiliations:** Laboratorio de Inmunología de Hongos, Unidad de Micología, Departamento de Microbiología-Parasitología, Facultad de Medicina, Universidad Nacional Autónoma de México (UNAM); Circuito Interior, Ciudad Universitaria, Av. Universidad 3000, México, D.F., 04510 Mexico; Laboratoire Biologie et Diversité des Pathogènes Eucaryotes Emergents, CIIL Institut Pasteur de Lille, Bâtiment Guérin, 1 rue du Professeur Calmette, Lille, France

**Keywords:** Surfactant proteins, Collectins, Respiratory fungal pathogens, Innate immune response

## Abstract

Pulmonary surfactant is a complex fluid that comprises phospholipids and four proteins (SP-A, SP-B, SP-C, and SP-D) with different biological functions. SP-B, SP-C, and SP-D are essential for the lungs’ surface tension function and for the organization, stability and metabolism of lung parenchyma. SP-A and SP-D, which are also known as pulmonary collectins, have an important function in the host’s lung immune response; they act as opsonins for different pathogens via a C-terminal carbohydrate recognition domain and enhance the attachment to phagocytic cells or show their own microbicidal activity by increasing the cellular membrane permeability. Interactions between the pulmonary collectins and bacteria or viruses have been extensively studied, but this is not the same for fungal pathogens. SP-A and SP-D bind glucan and mannose residues from fungal cell wall, but there is still a lack of information on their binding to other fungal carbohydrate residues. In addition, both their relation with immune cells for the clearance of these pathogens and the role of surfactant proteins’ regulation during respiratory fungal infections remain unknown. Here we highlight the relevant findings associated with SP-A and SP-D in those respiratory mycoses where the fungal infective propagules reach the lungs by the airways.

## Background

Pulmonary surfactant is a complex fluid that is composed of phospholipids (90 %) and proteins (10 %). There are four surfactant proteins (SP-A, SP-B, SP-C, and SP-D); each one has different biological functions. SP-A and SP-D are hydrophilic, whereas SP-B and SP-C are hydrophobic. SP-B, SP-C, and SP-D are essential for the lungs’ surface tension function and are required for the organization, stability and metabolism of lung parenchyma [1]. SP-A and SP-D are known for their contribution to the host’s lung immunity. Due to the role of SP-A and SP-D in the immune response, they have been preferentially studied in infectious diseases. The aim of this paper was to review the available information concerning the correlation between the surfactant proteins SP-A and SP-D and those respiratory fungal pathogens whose airborne infective propagules arrive at the lungs producing a progressive pulmonary disease.

## Review

Surfactant proteins are produced by different cell types, and in the lung, the four types are synthesized by type II pneumocytes [1]. SP-A and SP-D are also secreted by non-ciliated bronchiolar cells, submucosal gland and epithelial cells of other respiratory tissues, such as the trachea and bronchi. In the lacrimal apparatus, ductal epithelial cells are responsible for their production [2, 3]. Mucosal and glandular/ductal epithelial cells in the gastrointestinal tract (salivary glands, esophagus, small intestine, colon, pancreas, liver, and mesentery) produce low levels of SP-A and SP-D [2, 3]. These proteins have been found in sebaceous and sweat glands, where they are synthesized by the ductal and glandular epithelium; they are also found in the skin, but there is no evidence of surfactant proteins’ RNA transcripts in cutaneous cells [3]. SP-A and SP-D can be detected in both the male and female genitourinary tracts (prostate, testis, bladder, kidney, and uterus, even in non-pregnant women), and are apparently secreted by glandular and epithelial cells [2, 3]. Significant levels of SP-D, which has an important pro-atherogenic potential, were found in heart and brain tissues, where they are produced by vascular endothelial cells [3].

The chemical structure of SP-A and SP-D comprises different subunits: the N-terminal non-collagenous domain, a collagenous region, the helical neck, and the C-terminal carbohydrate recognition domain (CRD), each one with different ligand binding affinities [3].

The genomic locus of human SP-A consists of two functional genes, *SFTPA1* (*SP-A1*) and *SFTPA2* (*SP-A2*), and a pseudogene. This locus is situated on the long arm of chromosome 10 and the two functional genes are in opposite transcriptional orientation [4–6].

All mammalian species studied to date have a single-copy gene, except from primates onward, where a gene duplication occurred that gave rise to *SP-A1* and *SP-A2* genes; a recent report revealed additional SP-A sequences in some species, such as the opossum (three genes) and the chicken (*SP-A* and *SP-A*-like gene) [4]. A number of alleles have been characterized for each *SP-A* gene. The most commonly observed alleles for the *SP-A1* gene are *6A*, *6A2*, *6A3*, and *6A4*, and those for the *SP-A2* gene are *1A*, *1A0*, *1A1*, *1A2*, *1A3*, and *1A5*. Splicing variation and/or polymorphisms at the 59 and 39 non-translated regions of these alleles, respectively, point to regulatory differences [5].

Genetic studies have been conducted in human populations of adults, children, and newborns, where single nucleotide polymorphisms, haplotypes, and other genetic variants of *SP-A1* and *SP-A2* genes have been associated with acute and chronic lung diseases, like cystic fibrosis, asthma, allergic rhinitis, and chronic obstructive pulmonary disease [6]. Moreover, these human genetic variations have been considered risk factors for infectious diseases such as tuberculosis [7–9] and, for fungal infections in particular, there are *SP-A1* and *SP-A2* polymorphisms related to allergic bronchopulmonary aspergillosis [10].

The human SP-D locus is linked to the SP-A locus and is located proximal to the centromere at approximately 80–100 kb from the *SP-A2* gene. Several polymorphisms for SP-D and an association between SP-D alleles and lung diseases have also been identified [5].

SP-A and SP-D are classified in the C-type lectin family, commonly known as “collectins”, because of their structure. Collectins have a relatively high affinity for oligosaccharides, which suggests that they are important determinants of self/non-self recognition [11]. SP-A preferentially attaches only to monosaccharides, in the following order of binding preference: mannose, fucose, glucose, galactose, and N-acetylglucosamine; in contrast, SP-D binds more avidly to maltose, glucose, mannose, fucose, galactose, lactose, glucosamine, and N-acetylglucosamine [12], and to complex carbohydrates on the surface of different cells [13, 14]. SP-A stimulates the expression of the mannose receptor and the scavenger receptor A on the surface of alveolar macrophages, but these mechanisms are not clear [15].

SP-A and SP-D bind different pathogens, such as viruses, bacteria, fungi [6, 16, 17], and the nematode *Schistosoma mansoni* [18], via their CRD, and act as opsonins to trigger the mechanisms of the host’s innate immune response or, in some cases, they display their own microbicidal activity by increasing the permeability of the pathogen cellular membrane [1, 3, 15]. Overall, SP-A and SP-D opsonized-microorganisms (bacteria and fungi) enhance their attachment to phagocytic cells (macrophages and neutrophils), with subsequent pathogen clearance [3, 11].

Some interactions between collectins of the host’s defense mechanisms and the pathogen are known. Pulmonary collectins regulate cytokine and free radical productions, according to their environment, and play a pro- or anti-inflammatory role [3, 11]. Surfactant collectins inhibit T cell proliferation. In particular, SP-A inhibits dendritic cell maturation and SP-D stimulates antigen presentation by dendritic cells [15]. Furthermore, SP-A and SP-D possess direct bactericidal and fungicidal activity, but the exact mechanisms involved are still unknown [3, 11, 19]. It was described that SP-A decreases the binding of bacterial lipopolysaccharide (LPS) to the LPS-binding protein (LBP), acting as a competitive inhibitor and avoiding the initiation of the inflammatory cascade of LBP/CD14 [20]. However, Gardai et al. [14] demonstrated the increase of proinflammatory mediators of the innate and adaptative immune responses through the induction of nuclear transcription factors, kβ (NFkβ) and activator protein-1 (AP-1), by using LPS-stimulated macrophages after SP-A or SP-D attachment via their collagen regions to calreticulin/CD91 receptor complex on the host’s macrophage surface. In contrast, when the globular heads of these collectins bind to host’s macrophages, an anti-inflammatory effect is generated through a signal inhibitory regulatory protein α (SIRPα) that activates the tyrosine phosphatase-1 (SHP-1), which blocks src-family kinases and P38 MAP kinase signaling, therefore suppressing proinflammatory mediators [14]. These dual inflammatory functions of SP-A and SP-D could participate in the host’s immunological surveillance mechanisms for different microorganisms.

The interaction of surfactant proteins with Toll-like receptors (TLRs) and TLR-associated molecules, such as CD14, may be one mechanism for their inflammatory mediator function [11]. SP-A binds to the extracellular domain of TLR2 via its neck domain. The incubation of a recombinant soluble form of TLR2 with SP-A reduced the binding to bacterial peptidoglycan and the activation of NFkβ as well as TNF-α secretion by rat alveolar macrophages and U937 cells (a macrophage-like cell line) [21]. This finding suggests that direct interaction of SP-A with TLR-2 alters peptidoglycan-induced cell signaling, which results in a decreased inflammatory response [15]. In contrast, SP-A also associates with MD-2, a TLR-4 accessory protein and signaling molecule, and inhibits the binding of LPS to TLR-4/MD-2 in HEK293 cells (a human embryonic kidney cell line), which would activate NFkβ whether LPS had been attached to the TLR-4/MD-2 receptor complex. Therefore, SP-A inhibits NFkβ activation, explaining an anti-inflammatory mechanism previously described in vivo [15]. In regard to SP-D, it binds to the extracellular domains of TLR-2 and TLR-4 through its CRD by a different mechanism from those used in regular binding to pathogen phosphatidylinositol and LPS [22].

In regard to the role of SP-A and SP-D surfactant proteins in chronic lung diseases, including those produced by pathogens, is unknown. However, it has been suggested that the SP-D levels, in bronchoalveolar lavage fluid and serum, can vary substantially in different pulmonary conditions, which encourages the use of this protein as a biomarker of lung disease or injury [3, 11].

### Interaction of SP-A and SP-D with cytokines involved in the defense to pathogens

The collectins SP-A and SP-D have an important participation in the host’s innate immune response, where they interact with inflammatory cells and cytokines. This relationship has been described in different situations, such as immunoregulation during pregnancy [3], lung transplant rejection [23], pulmonary apoptotic cell clearance [3], allergic responses produced by pollen or *A. fumigatus* [3], and chronic inflammatory diseases [3, 24].

SP-A regulates macrophage function by diminishing the kinase activity required for the production of proinflammatory cytokines; it also upregulates the expression of IL-1β receptor, related to M-kinase (TLR-4 downregulator). Thus, SP-A inhibits TNF-α and IL-6 production, which is unleashed as a response to LPS [25]. In *Pseudomonas aeruginosa* infection, SP-A knockout mice show lower levels of IL-1β than do wild-type mice, which suggest that SP-A induces IL-1β production through the inflammasome pathway [26].

SP-A upregulates TLR-2 and TLR-4 transcription and post-translational modifications during monocyte-macrophage differentiation and downregulates Ikβα factor (NFkβ controller), which results in diminished TNF-α secretion in response to the binding of TLRs to their respective ligands [27].

SP-A plays a critical role in the differentiation and production of T regulatory cells (Treg), as described in SP-A deficient mice, which revealed an altered Foxp3 expression and the reduced production of Treg CD25 ^+^ Foxp3^+^ cells. SP-A also augments lL-2 and TGF-β levels [28].

In regard to SP-D, an anti-inflammatory effect mediated by IL-8 was detected after its binding to pollen particles, because this interleukin inhibits histamine discharge from basophils, thereby antagonizing IgE production in plasma B cells [29].

SP-D can compete with TNF-α in the proinflammatory effects on macrophages and dendritic cells by inhibiting partially this cytokine’s production; thus, TNF-α contributes to an indirect increase in SP-D by inducing IL-13 [30].

It has been described that SP-D knockout mice infected with *C. neoformans* displayed less eosinophil infiltration and lower IL-5 levels in bronchoalveolar lavage fluid than wild-type mice, suggesting a direct relationship between the presence of this collectin and the response to this pathogen [31].

### Interaction of collectins with respiratory fungal pathogens

The binding of SP-A and SP-D to a variety of opportunistic fungi results in the direct inhibition of fungal growth and the enhancement of phagocytosis [3]. However, the downstream immune response elicited by surfactant proteins can also contribute to the establishment of fungal infection and pathogenesis [3]. There are other medically important opportunistic fungi that cause pulmonary infections, such as *Candida* spp., which was not considered in this review because it enters the lung via hematogenous dissemination instead of the airway.

#### *Aspergillus fumigatus*

SP-A and SP-D surfactant proteins attach to *A. fumigatus* conidia via a calcium-dependent receptor, which increases phagocytosis and fungal destruction by neutrophils and alveolar macrophages [3, 32]; the fungal ligands recognized by SP-A include two N-glycosylated glycoprotein antigens (gp45 and gp55 kDa) secreted by *A. fumigatus*, which have been recovered from culture filtrates and used for immunodiagnosis of aspergillosis [32].

In a murine model of invasive pulmonary aspergillosis the intranasal administration of exogenous SP-D, either the purified human protein or a recombinant fraction containing the homotrimeric neck and the CRD domains (rSP-D), protected immunosuppressed mice from a fatal inoculum of *A. fumigatus* conidia [33]. This same experimental model was used by Singh et al. [34] and, according to their findings, the addition of native or recombinant SP-D reduced the fungal growth and increased the TNF-α and IFN-γ levels in murine lungs. SP-D gene-deficient mice are more susceptible to invasive pulmonary aspergillosis, whereas SP-A gene-deficient mice acquire resistance to this disease [35], which suggests that SP-A may facilitate the pathogenesis induced by *A. fumigatus*. Genetic studies show that mutations in the *SP-A2* gene (*G1649C*, *A1660G*, and the *AGA* allele) may increase susceptibility to *Aspergillus*-mediated allergies [10, 36].

#### *Blastomyces dermatitidis*

SP-D binds the β-glucan on the surface of *B. dermatitidis*, thereby blocking the access of β-glucan-receptors on alveolar macrophages to this molecule, which inhibits TNF-α production. *B. dermatitidis* may utilize SP-D as a strategy to reduce the host defense inflammatory reaction by diminishing TNF-α stimulation; which could favor the development of the disease [3, 37].

#### *Coccidioides posadasii*

SP-A and SP-D bind to coccidiodal antigens, but *C. posadasii* infection disrupts the expression of the pulmonary collectins, potentially enabling disease progression and promoting fungal dissemination [3, 38]. The levels of pulmonary surfactant collectins and phospholipids (measured by ELISA and Stewart method, respectively) were decreased in the lungs of mice infected intranasally with a lethal dose of *C. posadasii*; however, the collectins and phospholipid levels were normal in the lungs of *C. posadasii*-protected mice after immunization with a formalin killed spherule vaccine [38]. This study also assessed the concentration-dependent binding of SP-A and SP-D to coccidioidal antigens in vitro, and the findings sustained that pulmonary collectins were involved in the phagocytosis of *C. posadasii* by antigen presenting cells and in the downstream immune regulation of the infected host. Regarding the surfactant phospholipids, there is not sufficient information about their role of in the host defense [38].

#### *Cryptococcus neoformans*

SP-A binds to both encapsulated and non-encapsulated *C. neoformans* yeasts depending on the fungal concentration, but it does not enhance acapsular *C. neoformans* phagocytosis; this binding is calcium-dependent and can be inhibited by mannose and glucose, but not by galactose [39]. In a study on SP-A deficient and wild-type mice using an intranasal *C. neoformans* infection model, SP-A did not influence disease progression [40]. In contrast, SP-D agglutinates acapsular yeasts with a higher affinity than SP-A [41, 42], increases the phagocytosis of hypocapsular *C. neoformans* by murine macrophages under in vitro and in vivo conditions [43, 44], enhances fungal survival [44], and protects *C. neoformans* against oxidative stress in an experimental murine model, which facilitates the disease progression [43]. The ligands identified for SP-D are the capsular components glucuronoxylomannan and mannoprotein1 [42].

#### *Histoplasma capsulatum*

Only a few studies have described the particular interaction of *H. capsulatum* and surfactant proteins. McCormack et al. [19] showed that exposing *H. capsulatum* yeasts to SP-A and SP-D stimulated a dose-dependent decrease in [^3^H]leucine incorporation, as revealed by a failure to grow on Ham’s F12 supplemented medium. This exposure increased yeast permeability based on a leak of protein from the organism and enhanced the access of an impermeable substrate of the intracellular alkaline phosphatase. This mechanism is calcium-dependent, because calcium binding produces conformational shifts in the CRD region of the collectins, thus exposing charged or hydrophobic protein molecules with biological functions, such as interacting with membrane phospholipids or other surface components, and disrupting membrane function [19]. However, SP-A and SP-D did not inhibit the growth of macrophage-internalized *H. capsulatum* yeasts [19]. Besides, in this study, the authors also assayed an intranasal infection with *H. capsulatum* yeasts in SP-A null mice, demonstrating that these mice were more susceptible to the infection than age-matched wild-type control mice. This difference was associated with an abrogation in the number of pulmonary CD8^+^ cells and a higher fungal burden in the lungs and spleen of SP-A null mice than the wild-type littermates [19]. Neither SP-A nor SP-D aggregated *H. capsulatum*, based on light microscopic inspection of the fungus incubated with these collectins for different periods of time. Besides, none of these collectins altered the phagocytosis process of this pathogen [19].

#### *Paracoccidioides brasiliensis*

After an extensive bibliographic search, we found no studies on the interaction between *P. brasiliensis* and SP-A or SP-D; the same was reported by Brummer and Stevens [16] in their review on collectins and fungal pathogens. Information on this matter remains to be investigated.

#### *Pneumocystis* sp

The CRD region of SP-A and SP-D bind the major surface glycoprotein (MSG) of *Pneumocystis* sp. [45], which is the predominant membrane protein, is rich in mannose residues, and attaches *Pneumocystis* to the alveolar epithelium; this is mediated by a time- and calcium-dependent mechanism and is competitively inhibited by mannosyl albumin [46].

*Pneumocystis* has a narrow relationship with its host and displays a host-specific interaction known as stenoxenism, which was first described by Gigiliotti et al. [47] in 1993, and confirmed by Demanche et al. [48] in 2001. Each *Pneumocystis* species infects a particular mammal species: *P. carinii* and *P. wakefieldiae* infect rats, *P. murina* infects mice, *P. oryctolagi* infects rabbits, and *P. jirovecii* infects humans. Thus, before 2001, *P. carinii* was the only species referred and privileged in several studies.

In rats, SP-D markedly accumulates during *P. carinii* pneumonia and binds to MSG on the surface of this fungus, which contains an N-linked carbohydrate chain that is rich in glucose, mannose, and N-acetyl-glucosamine. The binding site uses a mannose-type saccharide, which is a calcium-dependent mechanism, and is competitively inhibited by maltose > glucose > mannose > N-acetyl-glucosamine [49]. Dodecamers and other large arrangements of SP-D bind better to *P. carinii* than oligomeric arrangements [49].

SP-D additionally binds to fungal β-glucans present on *Pneumocystis’* cystic forms, which are potent stimulators of TNF-α release [49]. *P. carinii* has other lectin binding activity, thus raising the possibility that a surface lectin on the fungus may also interact with the N-linked glycosylation of SP-D and other collectins [49].

Interestingly, none of these binding mechanisms increase the phagocytosis of this pathogen [46, 49]. However, SP-D significantly promotes *P. carinii* self-association, which results in large aggregates of organisms that may exhibit impaired uptake by macrophages; this potentially represents a mechanism for evading the microorganism’s elimination by the host [49].

*Pneumocystis* infection results in major changes in the expression of all surfactant components [50]. The total protein content of bronchoalveolar lavage increases 10-fold during *Pneumocystis* pneumonia, which induces a 3-fold increase in the total alveolar SP-A and SP-D protein content, due to the increased expression (mRNA) and accumulation of these collectins in surfactant fluid in human [51, 52] and rat samples [53]. Moreover, *Pneumocystis* pneumonia decreases the total phospholipid levels [50]. Both collectins are known to be increased as a result of enhanced translation, constitutive release, and decreased clearance from the pulmonary fluid. Transgenic mice over-expressing IL-4, related to the Th2 cytokine response to *Pneumocystis* pneumonia, increase SP-D mRNA [50].

Aliouat et al. [53] used two corticosteroid-untreated animal models (rabbits and severe combined immunodeficiency [SCID] mice), which were intranasally inoculated with *P. carinii*, and explored the content of surfactant phospholipids and proteins in bronchoalveolar lavage. In SCID mice, the surfactant phospholipid/protein ratio remains low, whereas the parasite increases and pneumonia progresses. However, in rabbits, the surfactant phospholipid/protein ratio and parasite rates were inversely proportional, similar to the events observed in AIDS-related *Pneumocystis* pneumonia in humans; these changes were present prior to the establishment of pneumonia with *Pneumocystis*’ proliferation [53].

Some studies have reported reduced surfactant phospholipid levels of bronchoalveolar lavage either in rats infected with *P. carinii* [54] or in humans with pneumocystosis [55]. The phospholipid composition of bronchoalveolar lavage was also altered in *P. carinii* pneumonia, with a slight increase in the percentage of sphingomyelin and a reduced percentage of phosphatidylcholine. SP-A inhibits phospholipids secretion by type II pneumocytes and stimulates its clearance [53]. There was no difference in the lavage phospholipase A2 activity for the *Pneumocystis*-infected and control groups [54].

The comparison among four groups of individuals: healthy volunteers, *Pneumocystis*-negative HIV-positive patients, mild *Pneumocystis* pneumonia patients, and moderate-to-severe *Pneumocystis* pneumonia patients, showed a reduction in total bronchoalveolar lavage lipids in the *Pneumocystis*-positive groups [55], particularly in the diacylglycerol lipids, whose predominant source is the surfactant’s dipalmitoyl phosphatidylcholine. Furthermore, diacylglycerol is metabolized by phospholipase A2, which showed increased activity in moderate-to-severe *Pneumocystis* pneumonia (twice the level of the *Pneumocystis*-negative patients and 30-fold the normal levels) [55]. Despite the incremented activity of the phospholipase A2 enzyme, its metabolism products (monoacyl glycerols and fatty acids) were not increased; this could be explained by a dynamic cellular uptake and metabolism of lysolipids and free fatty acids or a reduced production of surfactant by alveolar type II cells. There was also a marked decrease in surfactant glycerophospholipid in patients with AIDS and *Pneumocystis* pneumonia, which suggests a potential role in the pathophysiology of this disease [55].

## Conclusions

Interactions between the pulmonary collectins and different microorganisms, such as bacteria and viruses, have been extensively studied, but this is not the same for fungal pathogens. There is a lack of information on SP-A and SP-D binding to fungal carbohydrates, their relation with immune cells for the clearance of these pathogens, and the regulation of these proteins during fungal infection.

Respiratory fungal pathogens represent one of the most diverse groups of study in terms of surfactant proteins. Many of these fungi have a very narrow geographical limit, where they cause an endemic disease, such as *B. dermatitidis* and *Coccidioides* sp. Others, like *C. neoformans*, enter the organism by the respiratory route, but the lung is not their target organ; thus, they have a different association with surfactant proteins.

However, some fungi (*H. capsulatum* and *Pneumocystis* spp.) share important characteristics, including a worldwide distribution, the form of infection (inhalation of particles), and the possibility of dissemination (particularly in immunosuppressed individuals). Interestingly, they have also been found together co-infecting humans [56] and other mammals [57], which suggest a broader assembly between the fungal pathogens and the surfactant proteins than what it is known. The last feature makes them a trending study topic.

Very scarce evidence is available about SP-A, SP-D and *H. capsulatum*, contrasting the data concerning the pulmonary collectins and *Pneumocystis* spp. However, despite the apparent understanding of the relation between the latter, there are information gaps that should be investigated, hence opening a broad research field.
